# Modifiable risk factors of prostate cancer: Insights from a hospital-based study

**DOI:** 10.18632/oncoscience.657

**Published:** 2026-04-28

**Authors:** Maya Kulshekar, Anuradha B. Patil, Rajendra B. Nerli

**Affiliations:** ^1^Department of Biochemistry, Jawaharlal Nehru Medical College, KLE Academy of Higher Education and Research, Belagavi, Karnataka; ^2^Department of Urology, Jawaharlal Nehru Medical College, KLE Academy of Higher Education and Research, Belagavi, Karnataka

**Keywords:** modifiable risk factors, prostate cancer, awareness, India, molecular mechanism

## Abstract

Introduction: Prostate Cancer (PCa) is the second most common cancer and the fifth leading cause of cancer among men worldwide. The prevalence of Prostate cancer is much lower in India and it is likely that the incidence of prostate cancer will show an increase in the future. Modifiable habits like smoking, tobacco chewing, alcohol consumption and others have shown to be associated with Prostate Cancer. All these variables show conflicting results in various studies. Hence more research on the various determinants of Prostate Cancer for the Indian population will help in reducing the burden of this disease through scientific and empirical approach. The present study aims to study association of various modifiable risk factors with Prostate Cancer and Benign Prostatic Hyperplasia.

Methods: 72 histologically proven prostate ‘Cancer Cases’ and 132 ‘BPH controls’ were included for the study after obtaining their Informed Consent. This was confirmed by performing the PSA (Prostate Specific Antigen Test), DRE (Digital Rectal Examination) test and histopathological investigations. Odds ratio was obtained to study the association of the risk factors.

Results: Our study revealed that Modifiable risk factors associated with decreased risk of Prostate cancer are Coffee intake and meat with an odds ratio of 0.355 (95% CI 0.175 to 0.72, *p* = 0.004) and 0.516 (95% CI 0.269 to 0.992, *p* = 0.047). No significant association was observed with other modifiable risk factors.

Conclusions: The present study concludes that an increased intake of coffee and meat may be linked to a reduced risk of developing prostate cancer.

## INTRODUCTION

The second most frequent type of cancer in males and the fifth largest cause of cancer-related deaths globally is Prostate Cancer (PCa) [1, 2]. Although the Prostate Cancer incidence in Indians is lower when compared to the Western population, it is expected that the number of PCa cases shall increase in the years to come [3, 4]. As per the Indian Cancer Registry 2020 data, PCa accounted for 3% of all cancers in India with close to 40,000 cases being reported annually and an increase in 30% of cases in the last 25 years. The data on the modifiable lifestyle risk factor of prostate cancer is limited for any population and debatable. Many studies have shown alcohol, smoking, tea, coffee, red meat and occupational hazards as associated risk factors for PCa [5–8]. There are fewer studies reported on the Prostate Cancer modifiable risk factors for the Indian population. The present study will summarize the association of these risk factors and their potential mechanisms involved in development or prevention.

## RESULTS

The participant’s demographics and distribution of habits are summarized in Table 1 and Table 2. The mean age was 72 (±9.2) years for cases and 65 (±8.46) years for controls. Study participants were majorly in the age group between 61–80 years (74%). Among the cases, there was an overwhelming majority (86%) of the participants from rural areas when compared to the urban population. (Table 1). Multivariate logistic regression analysis was performed to evaluate the association between variables and outcome. (Table 3). In the unadjusted analysis, tobacco use (OR = 2.63, 95% CI: 1.41–4.91, *p* = 0.002) and smoking (OR = 2.54, 95% CI: 1.38–4.66, *p* = 0.003) were significantly associated with higher odds of the outcome. Coffee intake (OR = 0.30, 95% CI: 0.15–0.58, *p* = 0.001) and meat consumption (OR = 0.40, 95% CI: 0.22–0.74, *p* = 0.003) were associated with significantly lower odds of the outcome. After adjusting for covariates, the associations for tobacco and smoking were attenuated and became statistically non-significant. However, coffee consumption remained strongly protective (adjusted OR = 0.36, 95% CI: 0.18–0.72, *p* = 0.004), and meat consumption also retained a protective effect with borderline significance (adjusted OR = 0.52, 95% CI: 0.27–0.99, *p* = 0.047). Farming occupation showed no significant association with the outcome in either unadjusted or adjusted models. Very small numbers in the contingency table, led to infinite or undefined Odds Ratio value for Tea (Table 3).

**Table 1:** Demographic data of the study participants: Age distribution and regional variation

Variable	Cases *n* (%)	Controls *n* (%)
*Age*		
*41–50*	1 (0.01)	6 (0.04)
*51–60*	10 (14)	23 (17.4)
*61–70*	18 (25)	63 (47)
*71–80*	35 (49)	34 (26)
*>80*	8 (11)	6 (0.04)
*Regional Variation*		
*Rural*	62 (86%)	65 (49%)
*Urban*	10 (14%)	67 (51%)

**Table 2:** Distribution of habits among cases and controls

Variables	Case (*n*)	Controls (*n*)
**Tobacco chewing**		
*Yes*	53	68
*No*	19	64
**Smoking**		
*Yes*	33	33
*No*	39	99
**Alcohol consumption**		
*Yes*	20	43
*No*	52	89
**Tea**		
*Yes*	72	123
*No*	0	9
**Coffee**		
*Yes*	15	62
*No*	57	70
**Meat consumption**		
*Yes*	23	71
*No*	49	61
**Farming**		
*Yes*	67	112
*No*	5	20

**Table 3:** Multivariate logistic regression of potential risk factors associated with prostate cancer

Variables	Unadjusted			Adjusted		
	OR	95% C.I (LL, UL)	*p*-value	OR	95% C.I (LL, UL)	*p*-value
**Tobacco**	No	Ref.			Ref.		
	Yes	2.625	(1.405, 4.907)	0.002^*^	1.935	(0.976, 3.837)	0.059
**Smoking**	No	Ref.			Ref.		
	Yes	2.538	(1.382, 4.664)	0.003^*^	1.533	(0.768, 3.062)	0.226
**Alcohol**	No	Ref.			Ref.		
	Yes	0.796	(0.423, 1.497)	0.479	1.13	(0.534, 2.392)	0.749
**Coffee**	No	Ref.			Ref.		
	Yes	0.297	(0.153, 0.577)	0.001^*^	0.355	(0.175, 0.72)	0.004^*^
**Meat**	No	Ref.			Ref.		
	Yes	0.403	(0.221, 0.736)	0.003^*^	0.516	(0.269, 0.992)	0.047^*^
**Farming**	No	Ref.			Ref.		
	Yes	2.393	(0.858, 6.674)	0.095	1.79	(0.554, 5.788)	0.331

^*^p = < 0.05.

### Molecular mechanism in carcinogenesis

Alcohol consumption causes Prostate carcinogenesis by several mechanisms however, metabolites of ethanol such as acetaldehyde are significant in driving the growth of cancer cells and raising the metastatic potential of tumours [6, 9]. The protective function of nutrients such as trans-vaccenic acid found in meat improves immune response to cancer [10]. On the other hand, red meat contains recognized carcinogens, such as polycyclic aromatic hydrocarbons (PAHs) and heterocyclic amines (HCAs) [11, 12].

Several studies suggest that the main antioxidants found in green tea referred to as catechins, (-)epigallocatechin-3-gallate (EGCG), (-)-epigallocatechin (EGC) are known to inhibit tumorigenesis and tumour progression, [13]. Other polyphenols such as Flavonoids have been shown to increase the mRNA expression of tumor suppressor genes, thereby suppressing carcinogenesis [14, 15]. Cafestol and Kahweol present in coffee have anti-cancer properties [16]. Coffee contains antioxidant compounds such as caffeine, phenolic compounds and melanoidins which show protective function [17]. In addition, they also exhibit other important mechanisms such as anti-inflammatory, DNA damage control, and transcriptional factors modulation which prevent tumour growth [18]. One of the major causes of various types of cancers is Smoking. The molecular mechanisms state that the mutation by tobacco-related carcinogens may cause mutations of cancer progression genes [19]. Other hypotheses involve hormone alterations, the proliferation of tumour angiogenesis, and immune suppression [20]. According to recent research, neutrophil extracellular traps, or NETs, may have a role in a number of pathophysiological diseases, including cancer [21]. It has been discovered that smoking cigarettes causes vascular injury in a number of organs, including prostate cancer, through endothelial damage, oxidative stress, chronic inflammation, and local hypoxia [22]. Smokeless tobacco -products undergo fermentation which produces nitrosamines, the potential carcinogens [23]. Other possible mechanisms include exposure to aldehydes and metals, which have been associated with inflammation and increased cell proliferation [24]. Tobacco chewing also enhances the levels of ROS and increases the expression of inflammatory markers such as IL-18 which has an important role to play in tumourogenesis. It is also known to diminish the DNA repair mechanism [25]. Several organophosphate insecticides cause aggressive forms of cancer due to their role in the progression of cancer rather than initiation [26]. Organochlorine pesticides are endocrine disruptors that trigger PCa development [27, 28]. In addition to specific pesticides, a farmer may be exposed to several other chemicals such as mononuclear aromatic hydrocarbons, polycyclic aromatic hydrocarbons from petroleum, gasoline engine emissions, acetic acid arsenic compounds, lubricating oils and greases, diesel engine emission and alkanes whose role need to be investigated [29].

## DISCUSSION

**Figure 1:**
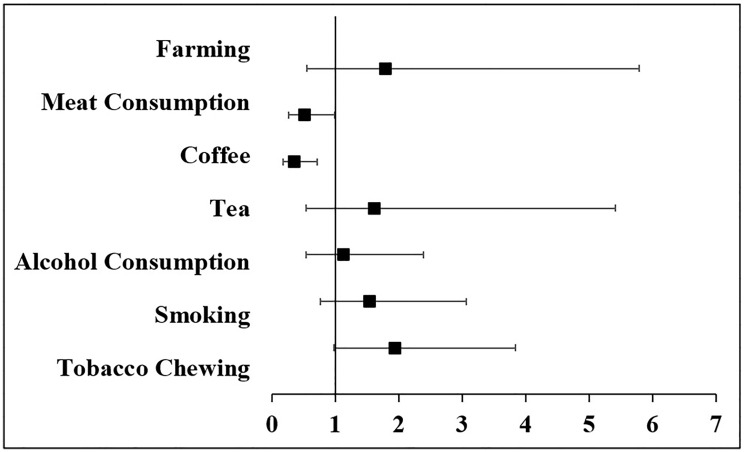
Odds ratio and corresponding 95% interval for the modifiable risk factors.

Modifiable Risk factors for prostate cancer have been well identified and supported by strong data. Some of these include the consumption of alcohol, tea, coffee, meat, smoking, tobacco chewing, and heavy physical activities such as farming have been discussed below. Of all the seven risk factors studied here, only coffee and meat consumption showed a significant association as depicted in Figure 1.

### Alcohol

The present study does not show an association between PCa and alcohol. This finding is comparable to another case-control study which was conducted by Ganesh et al. [5] however contradicts a study conducted in Delhi [30, 31]. Results from other studies have shown that with the increase in the number of doses, the risk of prostate cancer increases considerably [32]. A meta-analysis report suggested that alcohol consumption does increase the risk of morbidity and mortality [33]. A study conducted by Vartolomei et al. presented that PCa risk increases even with the consumption of white wine when compared to red wine [34]. However, among healthy subjects, a correlation was found between heavy alcohol use and a higher risk of dying from prostate cancer [35].

### Meat

In this study, meat consumption did show a significant association with PCa. This is supported by a similar study which stated that higher intakes of poultry 60 g/d and 30 g/d when substituted with red meat or unprocessed meat was linked to a noticeably lower incidence of recurrence [36].

A meta-analysis of the Asian population concluded that there is no association between prostate cancer risk and poultry consumption [37]. This is similar to a prospective meta-analysis which suggested that the consumption of red meat is not linked to prostate cancer [38]. Earlier studies do conclude that less meat consumption does not lower the chance of developing prostate cancer [39]. Contrary, an organized analysis study does indicate that higher prostate cancer risk is associated with “total meat” consumption [40].

Studies do suggest that replacing red meat with leaner proteins such as skinless poultry should be the diet of choice [41]. Studies have also concluded that a “plant source” diet unlike meat is protective against Prostate Cancer [42]. Our study restricted data collection to total meat consumption and not the kind of meat which should be taken up in future studies considering its significant association.

### Tea

Our research indicates no correlation between tea drinking and the incidence of prostate cancer. On the contrary, tea consumption in US states reduces the risk of PCa [43]. Our study also contradicts other studies which show an association between an increase in consumption of tea and a lower risk of Prostate cancer [44]. According to a meta-analysis, tea consumption could not bring a reduction in cancer and has additionally been demonstrated to raise the risk of prostate cancer [45, 46]. Further, the association of other types such as Green Tea is also not explored in our study which is shown to be protective in some studies [47, 48]. Being one of the common beverages consumed in India, the understanding between Tea consumption and its association with the development of cancer has always been unclear. From historical times, the consumption of tea is considered to be beneficial and preventive to the development of many diseases including Cancer. However, no clear statistical data is available [49].

### Coffee

Our study showed a 65% less chance of developing PCa with the intake of Coffee. A systematic review and meta-analysis on the intake of Coffee and PCa for a very large cohort has indicated that prostate cancer risk is lowered with increased intake of coffee [50, 51]. It is also comparable with another study which concludes that six or more cups of coffee per day, less the risk for lethal PCa [52]. A study conducted in the UK concluded that aggressive PCa risk is lowered with coffee consumption but not its overall risk [53]. Another meta-analysis for both cohort and casecontrol studies revealed that an increase in coffee intake may or may not decrease the risk of prostate cancer [54]. Our current study showed a significant association between the two. Hence, may be considered as a health recommendation to prevent the disease.

### Smoking

No positive association between smoking and PCa incidence was observed in a large cohort meta-analysis study [55]. This is comparable to another similar study which concludes that there is a lower risk of prostate cancer in smokers [56]. A study by Lora et al. indicated that smoking may be moderately associated with an increased risk of PCa [57]. This is contradictory to another study that reveals smoking has a lower risk for PCa [58]. Mortality and aggressive PCa forms also show an association with smoking in some studies [59]. Smoking is an established causative agent for cancer mortality in India, despite that our present study shows no association between smokers amongst prostate cancer. Our study also did not consider the kind of smoking or doseresponse and prostate cancer incidence, which may be intervened in the future, studies.

### Tobacco chewing

In rural India close to 40% of cancer patients still consume tobacco leaves and other tobacco products [60]. About 28 known carcinogens have been identified in smokeless tobacco [61].

Several prospective and retrospective studies have implicated the role of smokeless tobacco and the development of various cancers [62–64]. Tobacco chewing is one of the most common practices in this part of the country. Globally, India ranks third in production of tobacco and consumes 50% of it [65]. Numerous research studies have linked tobacco and its product use to a variety of deadly malignancies. However, our study has found to have no possible association between chewing and prostate cancer.

### Farming

A wide array of activities are involved in farming that enhance the exposure to potentially diverse agents such as pesticides, and insecticides that increase the risk of PCa [29, 66, 67]. Several Case-control and mortality studies are comparable with each other concerning their positive association with prostate cancer [68–70]. In another study by Meyer et al. on Caucasians, exposure to various farming activities showed an association with an increased risk of prostate cancer [71]. This finding is similar to the study conducted by Krstev et al. [72]. The use of pesticides in farming is one of the most common occupational hazards responsible for prostate carcinogenesis [71]. Our study did not show any association and does not have information on the kind of farming practices and type of chemicals the farmers are exposed to which can be evaluated in future studies.

## MATERIALS AND METHODS

### Study population

The present observational cross-sectional study was conducted at a tertiary care hospital in the city of Belagavi between 2020 and 2022. The data was analysed thereafter. An approval from the institutional ethics committee was secured and a total of 72 Prostate Malignancy cases (outcome of interest) and 132 Benign Prostatic Hyperplasia cases (controls) were included in our study.

The association of seven modifiable lifestyle risk factors was studied in each group.

### Data collection and statistical evaluation

All patients admitted to the Department of Urology as confirmed cases of Prostate Cancer and BPH were included in the study. After obtaining the Informed Consent of the patients, they filled out the questionnaire. All questions included in the questionnaire were single-choice questions. Data was entered into the SPSS software (version 20.0). Multivariate Logistic Regression analysis was completed to understand the association and odds ratios were estimated.

## CONCLUSIONS

The current study concludes that meat and coffee are the protective factors. Hence more studies must be taken up to understand the underlying mechanism and bioactive compounds present in them. In this study, most of the cases were from rural areas unlike what has been reported in cancer registries which were based on urban population. Although no association was seen between PCa, smoking and alcohol consumption. The data obtained here may not be relevant due to social discrimination and stigma involved with the perception of these two risk factors. The primary objective of the study was to assess overall consumption frequency rather than detailed dietary characterization which is not addressed here. Our study does have major limitations such as a being a hospital based study which limits the generalizability of results to broader community setting. Results from individuals who seek hospital care such as BPH and Prostate Cancer patients may vary systematically from the general population. Other limitations include recall bias, smaller sample size, inclusion of a few risk factors, non-inclusion of dietary dose-response, and exposure time of the risk factors which may be overcome by prospective preclinical studies and animal model studies. Future studies incorporating local consumption patterns in larger, multicenter prospective cohorts may be taken up to confirm associations. Developing countries like India have medical resources and hence can formulate strategies that prevent the disease by creating awareness among the population.

## Abbreviations

PCa: Prostate Cancer
BPH: Benign Prostatic Hyperplasia
PSA: Prostate Specific Antigen Test
DRE: Digital Rectal Examination
